# Non-coding RNAs in chromatin disease involving neurological defects

**DOI:** 10.3389/fncel.2014.00054

**Published:** 2014-02-25

**Authors:** Floriana Della Ragione, Miriam Gagliardi, Maurizio D'Esposito, Maria R. Matarazzo

**Affiliations:** ^1^Functional Genomics and Epigenomics Laboratory, Institute of Genetics and Biophysics “ABT,” Consiglio Nazionale delle RicercheNaples, Italy; ^2^Laboratorio di Genomica e di Epigenomica, Istituto di Ricovero e Cura a Carattere Scientifico NeuromedPozzilli, Italy

**Keywords:** Rett syndrome, ICF syndrome, non-coding RNA, chromatin, neurological desease, imprinting

## Abstract

Novel classes of small and long non-coding RNAs (ncRNAs) are increasingly becoming apparent, being engaged in diverse structural, functional and regulatory activities. They take part in target gene silencing, play roles in transcriptional, post-transcriptional and epigenetic processes, such as chromatin remodeling, nuclear reorganization with the formation of silent compartments and fine-tuning of gene recruitment into them. Among their functions, non-coding RNAs are thought to act either as guide or scaffold for epigenetic modifiers that write, erase, and read the epigenetic signature over the genome. Studies on human disorders caused by defects in epigenetic modifiers and involving neurological phenotypes highlight the disruption of diverse classes of non-coding RNAs. Noteworthy, these molecules mediate a wide spectrum of neuronal functions, including brain development, and synaptic plasticity. These findings imply a significant contribution of ncRNAs in pathophysiology of the aforesaid diseases and provide new concepts for potential therapeutic applications.

## Introduction

Our common view of the complexity of mammalian transcriptome has been revolutionized by the advent in the high-throughput sequencing highlighting that tens of thousands of sites produce transcripts with tiny protein-coding potential. Indeed, it is now increasingly clear that the vast majority of the genome is transcribed in both sense and antisense directions and this transcription is active in a cell context- and a developmental stage-specific way. Moreover, a rigorous distinction between protein-coding and non-coding transcripts is misleading, considering that some non-coding RNAs (ncRNAs) can be translated and other RNA transcripts may participate in regulatory and functional processes, rather than serving simply as intermediates for translation.

**Figure 1:**
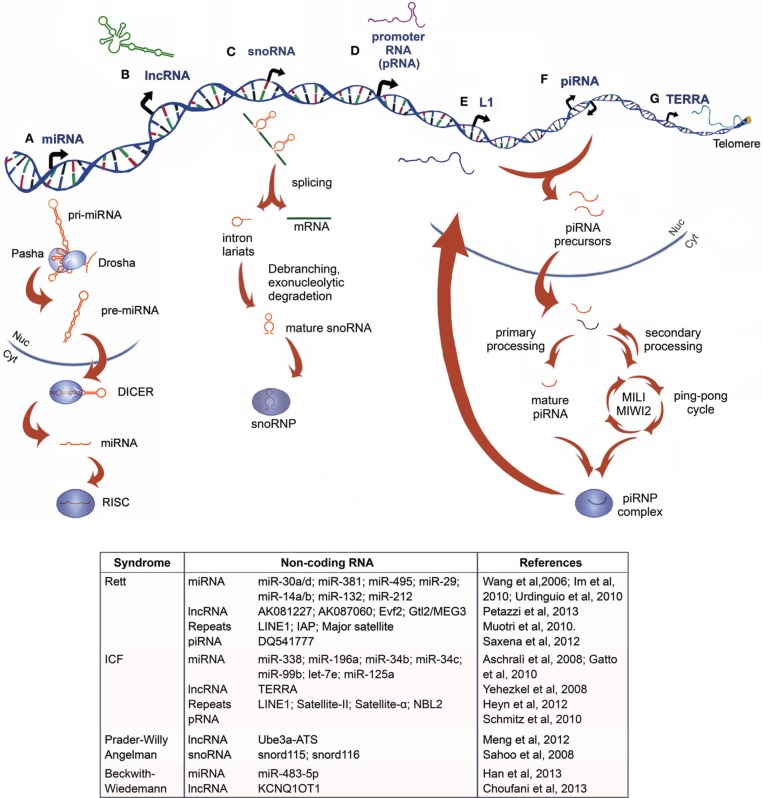
**Non-coding RNAs: biogenesis and their relation to chromatin disorders. (A)** MicroRNAs originate as primary miRNA (pri-miRNAs) that are processed by the DROSHA/PASHA complex in the nucleus. The resulting precursor miRNAs (pre-miRNAs) are exported into the cytoplasm, where they are processed by DICER1 to form mature miRNAs, which interact with RNA-induced silencing complex (RISC), acquiring a post-transcriptional silencing activity **(B)** Long non-coding RNA are heterogeneous transcripts, often longer than 2 kb, transcribed from intra- and intergenic regions by RNApol-II and, rarely, RNApol-III **(C)** Small nucleolar RNAs (snoRNAs) are codified primarily from introns. After the pre-mRNA generation, the transcript is spliced and the intron lariat is debranched and trimmed. The mature snoRNA interacts with ribonuclear proteins (RNPs), before being exported into the cytoplasm with a role in ribosomal RNA (rRNA) modification and processing, or retained into the nucleus, where it plays a role in alternative splicing and other unknown functions **(D)** In mouse, promoter-RNAs (pRNAs) are generated from intergenic regions located around 2 kb upstream to the pre-rRNA transcriptional start site. They are processed in molecules of 150–250 nucleotides and are thought to form a DNA:RNA triplex at the promoter, from which they are transcribed **(E)** Long interspersed nuclear elements-1 (LINE-1 or L1s) are retrotransposons dispersed throughout the genome that can be transcribed and inserted as extracopies of themselves **(F)** Piwi-interacting RNA (piRNA) originates as precursors from transposons and large piRNA clusters, then they are processed and exported into cytoplasm, where they undergo a primary or cyclic secondary processing (ping pong cycle, mediated by PIWI, MIWI, and MIWI2 proteins in mouse) and the assembly in piRNP complexes that modulate transposon activity and gene expression **(G)** Telomeric repeat-containing RNA (TERRA) is a large non-coding RNA transcribed from chromosome ends directly involved in the telomeric heterochromatin organization and preservation of telomere length. An up-to-date summary of the diverse class of ncRNAs, which have been functionally associated to Rett Syndrome and ICF Syndrome, as well as to the imprinting disease Prader-Willy/Angelman and Beckwith–Wiedemann syndromes, is reported in the table.

NcRNAs are commonly classified based on their size in two major groups: small ncRNA (sncRNAs, 20-30nt) which are involved in post-transcriptional regulation of target RNAs via RNAi, and/or modifying other RNAs, including microRNAs (miRNAs), Piwi-interacting RNAs (piRNAs) and small nucleolar RNAs (snoRNAs), and the heterogeneous group of long ncRNAs (lncRNAs, >200nt), such as transcribed ultraconserved regions (T-UCRs) and large intergenic ncRNAs (lincRNAs) (Figure 1).

MiRNAs are a class of small ncRNA with a role in post-transcriptional gene regulation through the base-pairing with the 3'-UTR of the target transcript. This interaction leads to degradation of the target or to inhibition of translation (Kim et al., 2009). siRNAs and piRNAs are small ncRNAs generated through the processing of LINE-1 and other retrotransposons, being involved in the silencing of repetitive elements (Saxena et al., 2012).

LncRNAs have roles in transcriptional and epigenetic regulation by recruiting transcription factors and chromatin-modifying complexes to specific nuclear and genomic sites (Khalil et al., 2009); in alternative splicing and other post-transcriptional RNA modifications through the assembly of nuclear domains containing RNA-processing factors (Wang and Chang, 2011; Caudron-Herger and Rippe, 2012). The emerging structural and functional activities of lncRNAs were categorized within a well-designed framework as molecules of signals, decoys, guides, and scaffolds (Wang and Chang, 2011).

## Non-coding RNAs in neuronal functions

NcRNAs are involved in mediating a broad spectrum of biological processes especially those occurring in the central nervous system, where neural cells are highly expressing diverse classes of ncRNAs. Indeed, ncRNAs provide neural cells with the ability to precisely control the spatio-temporal expression of genes, which is a critical aspect for fulfilling complex neurobiological processes. The newest finding that in neural tissue mRNAs express significantly longer 3'UTRs, compared to other tissues, provides intriguing mechanisms for cell type-specific regulations that are governed by miRNAs, RNA-binding proteins and ribonucleoprotein aggregates (Wang and Yi, 2014).

For instance, various miRNAs show selective expression or are particularly abundant in brain (Kosik, 2006). Indeed, DICER ablation in mouse cerebral cortex, interrupting the biogenesis of all miRNAs, reduces the cortical size, by decreasing neural stem cell and neural progenitor pool, increasing apoptosis and affecting neuronal differentiation, whereas its ablation in postmitotic neurons in the cortex and hippocampus results in smaller cortex and increased cell death (Bian and Sun, 2011). Specific miRNAs have been implicated in neuronal differentiation and maintenance of neuronal phenotype (Im and Kenny, 2012), being integrated with the circuitry of CREB, repressor-element-1 (RE1)-silencing transcription factor REST and REST-corepressor 1 (CoREST), which are master transcriptional and epigenetic regulators of neural genes and neural cell fate decisions (Qureshi and Mehler, 2012).

While most efforts have been devoted in identifying the neurobiological functions of miRNA, studies characterizing the expression and function of other classes of small and long ncRNAs in the central nervous system has begun to emerge. For instance, a specific set of piRNAs is highly expressed in the hippocampus, where they might play a role in spine morphogenesis (Lee et al., 2011). LncRNAs are also likely to have important roles in shaping brain development (Mercer et al., 2008; Ponjavic et al., 2009). A functional study examined the chromatin-state signature of neural progenitor cells (NPCs) identifying around one thousand lincRNAs, with some of them associated with hippocampal development, oligodendrocytes maturation and GABAergic-neuronal function (Guttman et al., 2009). A large percentage of these lincRNAs are involved in recruiting chromatin-modifying complexes to their genomic target sites, serving as scaffolds to specify the pattern of histone modifications (Tsai et al., 2010). Moreover, MeCP2 physically associates with RNCR3 (retinal non-coding RNA 3), unveiling new mechanisms of MeCP2/lncRNAs-mediated chromatin remodeling (Maxwell et al., 2013).

These observations provide insight into the complex interplay between ncRNAs and multifunctional epigenetic and transcriptional regulatory complexes coordinating neuronal lineage specification. In line with that, it is expected that if these ncRNAs regulate the development and maintenance of healthy neurons, their dysfunction may contribute to neurodevelopmental abnormalities (Qureshi and Mehler, 2012).

Here, we focused on the latest findings linking ncRNAs into the molecular and neural phenotypical defects of human disorders with deficiencies in (i) epigenetic modifiers, like MeCP2 (methyl-CpG-binding-protein-2) for the Rett syndrome (RTT) and DNMT3B (DNA-methyltrasferase-3B) for the Immumodeficiency, Centromeric instability, Facial anomalies (ICF) syndrome (Type1) or (ii) in the establishment of DNA methylation signature, as for the imprinting disorders (Figure 1).

Mutations in MeCP2 gene are linked to the severe postnatal neurodevelopmental disorder RTT syndrome, the second most common cause of mental retardation in females. It is characterized by developmental stagnation and regression, loss of purposeful hand movements and speech, stereotypic hand movements, deceleration of brain growth, autonomic dysfunction and seizures (Chahrour and Zoghbi, 2007). The ICF syndrome is caused by genetic defects in the catalytic activity of DNMT3B protein leading to genomic DNA hypomethylation and heterochromatin defects (Matarazzo et al., 2009). Beside immunological abnormalities, patients exhibit from mild to severe mental retardation and neurologic problems, including slow cognitive and motor development and psychomotor impairment (Ehrlich et al., 2006).

Defective inheritance of the imprinted signature is the main cause of the Prader–Willi (PWS) Angelman (AS) and Beckwith–Wiedemann syndromes (BWS). Both AS and PWS show learning dysfunctions and behavioral defects. Mutations or loss of the maternally-expressed gene UBE3A are responsible for AS, whereas loss of paternally-expressed genes, probably including SNRPN, causes PWS. BWS is caused by altered expression of imprinted genes belonging to a gene cluster at 11p15.5, and patients exhibits neurodevelopmental defects (Kent et al., 2008).

## Non-coding RNAs as transcriptional and post-transcriptional regulators

RTT is primarily caused by mutations in MECP2 gene, codifying a protein involved in both transcriptional repression and activation. Mecp2-null mice show many neurological symptoms recapitulating RTT (Della Ragione et al., 2012). Until recently, the classical approach to search for MeCP2 target genes involved in Rett pathogenesis was focused on protein-coding RNA transcripts. However, recent findings suggest a role of MeCP2 in transcriptional regulation of different classes of ncRNAs.

In 2010, two different large-scale analyses highlighted global dysregulation of miRNAs in MeCP2-null mice. Wu and colleagues identified several up- and down-regulated miRNAs in cerebella of Mecp2-deficient mice and, for many of them a direct promoter binding of MeCP2 was demonstrated. Interestingly, MeCP2 regulates the expression of a large cluster of miRNAs embedded within the Dlk1-Gtl2 imprinting domain, showing different levels of deregulation, suggesting the existence of miRNA-specific posttranscriptional regulation. Remarkably, MeCP2 silences miR-30a/d, miR-381 and miR-495, which in turn repress Brain-derived neurotrophic factor (BDNF), the down-regulated target gene in Mecp2-null mice (Wang et al., 2006); this suggests a multilayered MeCP2-mediated transcriptional regulation of BDNF (Wu et al., 2010).

Similarly, microarray expression analysis of Mecp2-null brains revealed alterations of several miRNAs. Noteworthy, deregulated miR-29 and miR-146 are known to have roles in neural and glial cells and its association with neurological disorders is reported (Urdinguio et al., 2007).

Although the classical form of RTT is associated with loss-of-function MECP2 mutations, duplication of this gene is responsible for RTT-like neurological phenotypes (Del Gaudio et al., 2006); therefore, a fine-tuning of MeCP2 level might be important for normal development.

In the last years, some reports highlighted a miRNAs contribution to maintain the correct levels of MeCP2. In rat neurons homeostatic mechanisms regulate MeCP2 levels through the stimulation of BDNF expression, which in turn induces miR132, thereby silencing MeCP2 expression (Klein et al., 2007). Later, it was shown that miR-212, located in the same cluster of miR-132, also targets MeCP2 and that MeCP2 silences the expression of both miRNAs, confirming the existence of a similar negative homeostatic feed-back loop between MeCP2 and mir-212, (Im et al., 2010).

Although several works highlighted a role of MeCP2 in transcriptional regulation of miRNAs, its involvement in the control of lncRNAs was poorly investigated. In Mecp2-deficient cerebella increased levels of Gtl2/MEG3, an imprinted ncRNA specifically expressed from maternal allele has been described. MeCP2-direct binding to the differentially methylated region controlling maternal specific expression was reported, suggesting a role of MeCP2 in the repression of paternal allele in the cerebellum (Jordan et al., 2007).

Very recently, the comparison of MeCP2-null and wt mouse brains transcriptome profiles highlighted 701 deregulated lncRNAs. Among lncRNAs with neuronal functions, the authors validated the up-regulation of AK081227 and AK087060, whose promoter is directly bound to MeCP2 in wt. Interestingly, the up-regulation of AK087060 is associated with an overexpression of the host gene, Arhgef26, codifying a Rho-guanin-nucleotide-exchange-factor (GEF) involved in neuronal functions, whereas the up-regulation of AK081227 is associated to a down-regulation of its host gene, the GABA-receptor-subunit-rho-2 (Gabrr2). Noteworthy, the GABAergic inhibitory neurotransmission is impaired in RTT (Coghlan et al., 2012). These findings indicate that lncRNAs deregulation in a MeCP2-deficient context may contribute to the RTT pathophysiology providing evidence for disrupted cis-regulated mechanisms in the disorder (Petazzi et al., 2013).

Recently, a functional link between ncRNAs, MeCP2 and GABAergic interneuron development has been found. Embryonic ventral forebrain-2 (Evf2) lncRNA is transcribed from the conserved Dlx-5/6 intergenic enhancer and regulates Dlx5 and Dlx6 expression during forebrain development by trans- and cis-acting mechanisms, respectively. It was shown that Evf2 recruits both DLX2 (activator) and MeCP2 (repressor) on the same ultraconserved Dlx5/6 enhancers in order to balance the correct expression of Dlx5 and Dlx6 during brain development. Remarkably, Evf2 loss causes GABAergic interneurons decrease in developing hippocampus. GABA-dependent inhibitory cortical activity is impaired in RTT-mice, thus MeCP2 and Evf2 loss may share common mechanisms (Bond et al., 2009). More recently, it is shown that MeCP2 represses Evf2 and Dlx5, whose expression is stimulated by DLX1/2 and that Evf2 prevents the DNA methylation of Dlx5/6 enhancer regardless MeCP2 (Berghoff et al., 2013). Altogether, these results suggest that ncRNAs deregulation may contribute to RTT pathoetiology.

While MECP2 reads and interprets the epigenetic signature, the *de novo* DNMT3B is the epigenetic factor establishing it during development.

Mutations interfering with its catalytic activity are known to affect the transcriptional profile of several hundred protein-coding genes in ICF patients' derived cells, with enriched functional classes including development and neurogenesis (Jin et al., 2008). Most of them do not exhibit promoter methylation defects, meaning that they are indirectly affected by DNMT3B deficiency (Matarazzo et al., 2004). Interestingly, a significant proportion of these genes are target of miRNAs, which are inversely deregulated in ICF-derived cells, suggesting that miRNAs are integrated in the DNMT3B-mediated regulatory circuitry modulating cell-specific gene expression program (Gatto et al., 2010). MiR-338 is the most up-regulated miRNAs with brain-specific functions, while among the down-regulated miRNAs, miR-196a is transcribed from intergenic regions within Hox genes clusters in vertebrates. As for the Hox genes, miR-196a acts as regulators of the nervous system development in embryos (Kosik, 2006). Intriguingly, LHX2, which is crucial for the proper development of cerebral cortex in mouse embryo is a miR-196a target gene, and results overexpressed in DNMT3B-deficient cells (Gatto et al., 2010).

Beyond the pivotal role of DNA methylation in miRNAs transcriptional control, there are examples describing the effect of ncRNAs on the epigenetic machinery and the DNMTs. The finding that a promoter-associated RNA recruits DNMT3B, inducing the methylation and the following repression of ribosomal RNA genes (rDNA) reveals a fascinating mechanism of RNA-dependent DNA methylation in mammals (Schmitz et al., 2010). Interestingly, DNMT3B exhibits binding specificity for a DNA:RNA-triplex, which are structures contributing to promoter-specific transcriptional repression by compromising transcription factor binding. That raises the exciting possibility that triplex-dependent recruitment of DNMT3B to specific genes might be a common function of regulatory ncRNAs.

Ribosomal RNAs are the target of another class of ncRNAs, the small nucleolar RNAs. These molecules are intermediate-sized ncRNAs (60–300 nt) predominantly intronic, which might be exported for processing the ribosomal RNA and/or remain in the nucleus for alternative splicing of mRNAs through yet unknown mechanisms.

They have been found playing an important role in imprinting disorders, specifically those with a neurodevelopmental component, such as PWS and AS. They are caused by several genetic and epigenetic mechanisms involving the 15q11–q13 imprinted locus, which contains a cluster of tandemly-arranged snoRNAs (Sahoo et al., 2008). Loss of the snoRNA HBII-52 has been associated to PWS, in which it regulates the alternative splicing of the serotonin receptor HTR2C precursor mRNA, resulting in a protein with reduced function. Additional snoRNAs at 15q11.2, including the Snord116 cluster, are even more likely candidates for causing PWS (Duker et al., 2010).

The PWS imprinting-control region contains multiple neuron-specific ncRNAs, including the antisense transcript to AS-causing ubiquitin-ligase Ube3a (Ube3a-ATS) (Meng et al., 2012). Neuron-specific transcriptional progression through Ube3a-ATS correlates with paternal Ube3a silencing. Intriguingly, topoisomerase inhibitors represses Ube3a-ATS inducing formation of DNA:RNA hybrids (R-loops), thus reverting the paternal allele silencing and providing a means to compensate for the loss of maternal Ube3a in AS patients (Powell et al., 2013).

Beckwith-Wiedemann (BWS) syndrome is also associated with altered expression of imprinted genes at 11p15.5. In particular, BWS results from increased expression of the paternally-expressed growth promoter IGF2 and/or reduced expression (or loss of function) of the maternal growth suppressor CDKN1C. An intragenic miRNA of the imprinted IGF2 (miR-483-5p) regulates MeCP2 levels through a human-specific binding site in the MECP2 long 3'-UTR. There is an inverse correlation of miR-483-5p and MeCP2 levels in developing human brains and fibroblasts from BWS patients. Importantly, the expression of miR-483-5p rescues abnormal dendritic spine phenotype of neurons overexpressing MeCP2.

LncRNA appear to play a key role in the BWS phenotype. KCNQ1OT1 is paternally transcribed from the imprinted-domain-2 of chromosome 11p15.5 (Weksberg et al., 2001). In the majority of BWS, loss of maternal methylation at IC2 is associated with bi-allelic transcription of the KCNQ1OT1 maternal allele (Choufani et al., 2013) and the bi-allelic silencing of the imprinted-domain genes, including the cell growth inhibitor CDKN1C (Diaz-Meyer et al., 2003). Notably, CDKN1C loss-of-function causes the typical overgrowth and increased risk to develop embryonic tumor in BWS patients.

## Non-coding RNAs and transcriptional control of repeats and heterochromatin

Recent reports highlighted a widespread misregulation of repeat-elements in Mecp2-deficient contexts. In rodents, MeCP2 loss is responsible for alteration of LINE1 neuronal transcription and retrotransposition (Muotri et al., 2010). An increased activity of L1 promoter in mouse Mecp2-null NPCs correlates to increased transcription. Noteworthy, increased L1 retrotransposition was also observed in NPCs generated from RTT patients induced pluripotent stem cells (iPSCs) and in postmortem brains from RTT patients. Therefore, MeCP2 seems to control L1 mobility in the nervous system and the absence of a functional protein may deregulate the retrotransposition with potential consequences for RTT (Muotri et al., 2010).

Further evidences on MeCP2-mediated regulation of repetitive elements showed that L1 retrotransposons, intracisternal-A-particles (IAP) and the major-satellite are up-regulated in Mecp2-null brains. These findings suggest that MeCP2 may act as a global regulator, probably by suppressing the spurious transcription of repetitive elements, thereby reducing the transcriptional noise (Skene et al., 2010).

LINE1 processing originates small ncRNAs including piRNAs, with a function related to the retrotransposons silencing. Therefore, it can be supposed that in a context of LINE1 excess, piRNAs may be also deregulated. The level of piRNAs in wt and Mecp2-null mouse cerebella by Wu et al. (2010) has been examined, finding 357 piRNAs expressed in wt cerebellum, the 59% of them showing a marked up-regulation in Mecp2-null mice. Intriguingly, the DQ541777 piRNA, important for the dendritic spine size that is impaired in Rett syndrome, is also overexpressed in Mecp2-null mice. In this light, it was proposed a model in which, in the absence of functional MeCP2 the overexpression of LINE-1 may lead to an increase in the piRNAs levels affecting the expression of specific genes deregulated in RTT (Saxena et al., 2012).

An important process in which the epigenetic players MeCP2 and DNMT3B interact with ncRNAs is the maintenance of the integrity at telomeric heterochromatin to prevent the telomere shortening (Deng et al., 2010). Telomeric-repeat-containing-RNAs (TERRAs) are lncRNAs transcribed from telomeres, involved in the formation of telomeric heterochromatin through a negative-feedback looping mechanism. TERRAs interact with several heterochromatin-associated proteins, including MeCP2, HP1, and H3K9 methyltrasferase. This interaction may be sufficient to nucleate heterochromatin complexes; the recruitment of DNMT3B and the establishment of CpG methylation at subtelomeres likely mediate this process. Indeed, in ICF-derived-cells TERRA levels are abnormally increased and telomeres are abnormally shortened (Yehezkel et al., 2008). The proposed mechanism implies that loss of CpG methylation at subtelomeres due to DNMT3B mutations results in a failure of TERRA-mediated feedback-looping. This allows for transcription factors to access to subtelomeres leading to a permissive chromatin state. Highly induced TERRA levels may contribute to telomere dysfunction in ICF cells either by inhibiting telomerase activity, forming RNA-DNAs hybrids with telomeres, or by interactions with telomere-binding proteins (Deng et al., 2010). The latest live imaging-based model, in which TERRA sequesters and delivers telomerase to the chromosome end from which the transcript originated, support the first mechanism (Cusanelli et al., 2013).

Genome-wide studies have reported an extensive reduction of DNA methylation at several repeat families, with satellite and transposons revealing the highest reduction. That implies the DNMT3B activity as crucial for centromere stability and transposon repression (Heyn et al., 2012). Overexpression of satellite II and satellite α DNA at pericentromeric and centromeric heterochromatin respectively has been indeed reported (Alexiadis et al., 2007), which might be functionally involved in the chromosomal aberrations in ICF syndrome.

## Conclusions

Increasing reports highlighting aberrant regulation of different classes of ncRNAs in chromatin disorders characterized by neurological defects, open new perspectives for the comprehension of the pathogenetic mechanism of these disorders. These misregulated ncRNAs may be identified as responsible for specific subset of complex phenotypes, providing us with valuable means to the development of more effective therapeutic strategies based on RNA regulators.

Experimental approaches using high-throughput chemical screens are being proved to be suitable to find molecules that can both activate or inhibit the RNAi/miRNA pathway and, potentially correct some neurological defects (Li et al., 2010). Therapeutic strategies based on the design of specific oligonucleotides targeting ncRNAs, with the aim to specifically silence or replace the transcript, are being explored (Esteller, 2011) and appear very promising. However, their selective delivery into the CNS remains the most considerable challenge, and until now it has not been addressed effectively.

Noteworthy, recent reports indicated that miRNA expression profile in peripheral blood samples mirror the one present in the brain after cerebral ischaemia subsequent to stroke (Tan et al., 2009). In addition, neural cells can release ncRNAs in the blood and cerebrospinal fluid in membrane-bound exosomes (Skog et al., 2008). These evidences suggest that ncRNAs from body fluids might be used as biomarkers of neurological diseases. Further efforts are needed to decipher the intricate ncRNA world, and help to clarify how deregulation of ncRNAs may contribute to neuronal dysfunctions.

### Conflict of interest statement

The authors declare that the research was conducted in the absence of any commercial or financial relationships that could be construed as a potential conflict of interest.
